# Multimodal, Multiband, and Multiple Anticounterfeiting Devices with Angle-Dependent Structural Color Highly Sensitive to Temperature

**DOI:** 10.34133/research.0919

**Published:** 2025-10-24

**Authors:** Gang Li, Shancheng Wang, Huaxu Liang, Wei Luo, Huiru Ma, Yi Long, Jianguo Guan

**Affiliations:** ^1^ Wuhan Institute of Photochemistry and Technology, Wuhan 430083, P. R. China.; ^2^ State Key Laboratory of Advanced Technology for Materials Synthesis and Processing, School of Materials Science and Engineering, Wuhan University of Technology, Wuhan 430070, P. R. China.; ^3^ School of Materials Science and Engineering, Nanyang Technological University, Singapore 639798, Singapore.; ^4^ Wuhan Xinguang Technology Co., Ltd., Wuhan 430083, P. R. China.; ^5^ Department of Electronic Engineering, The Chinese University of Hong Kong, New Territories, Hong Kong SAR, P. R. China.

## Abstract

Conventional anticounterfeiting devices rely on high-resolution equipment and only provide limited encrypted information, which greatly constrain the efficacy of ever-increasing high-end counterfeiting functionalities. In this study, we propose a simple solution method to fabricate an arbitration anticounterfeiting device with multimodal, multiband, and multiple optical properties, where a thermoresponsive magnetic photonic crystal hydrogel film is in situ chemically anchored onto a modified glass and sealed with a gasket and single-sided indium-tin oxide glass. The resulting device exhibits distinguishable optical responses in terms of reflection, transmission, and emission modes, covering a wide range of wavelengths from visible to near-infrared and long-wave infrared. Notably, the wide-angle dependent structural color of the device may change in the full visible spectrum (red to blue) with temperature, and the sensitivity is up to 11.5 nm/0.1 °C with stable repeatability. This study presents a promising avenue for the design and development of high-end anticounterfeiting devices for advanced applications.

## Introduction

Counterfeiting remains a long-standing global issue with wide-ranging detrimental impacts on various products including banknotes, valuable documents, medicine, brands, and luxury and common consumer goods, thereby threatening social and economic life, human health, and national security, among others [1–4]. During the past decades, a variety of anticounterfeiting strategies have been developed, such as watermarks [5,6], barcodes and quick response codes [7–9], radio frequency identification [10,11], physical unclonable function [12,13], optical anticounterfeiting [14], and so on [15,16], which have provided certain countermeasures against the increasing fraudulent practices worldwide. Among them, optical anticounterfeiting holds the unique visual effects produced by optical phenomena such as scattering, reflection, transmission, absorption, and diffraction [17]. It is usually achieved by utilizing luminescent materials [18–20], optical variable devices [21,22], and responsive photonic crystals (PCs) [23–25]. In particular, the use of responsive PCs enables tunable optical properties, as their structural parameters including lattice constants, refractive indices, and glancing angles can be adjusted by external stimuli [26–28]. This makes them promising for enhancing security and authenticity verification [29]. However, the most common responsive PCs only show a single security feature (color change). They cannot meet the anticounterfeiting market requirements due to the sophisticated techniques employed by counterfeiters.

Recently, the integration of 2 security elements has been proposed to improve the effectiveness of optical anticounterfeiting. For example, geminate labels have been developed via programming fluorescent cholesteric liquid crystal microdroplets by integrating cholesteric liquid crystal with luminescent material (fluorescent molecule), which exhibit structural color and fluorescent color in reflection mode and fluorescent mode, respectively [30]. The anticounterfeiting film, fabricated by co-assembling chiral nematic cellulose nanocrystal, lanthanide complexes, and poly(ethylene glycol), displays similar structural colors and fluorescence color [31]. Multiple anticounterfeiting has been achieved by employing external stimuli to manipulate the structural colors and regulate photoluminescence simultaneously [32,33]. Nevertheless, achieving third-line anticounterfeiting measures (arbitration anticounterfeiting), which can only be identified by experts via using physical, chemical, and other principles with special instruments, remains challenging [34].

**Fig. 1.**
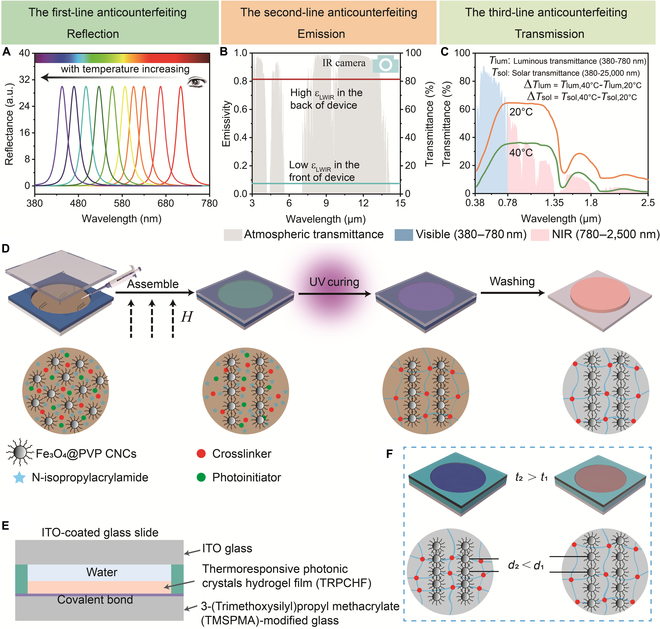
Concept and structure of multimodal, multiband, and multiple anticounterfeiting devices with high sensitivity. (A to C) Concept of the ideal multimodal, multiband, and multiple anticounterfeiting device. In reflection mode, temperature-induced color change can be recognized by the naked eye, serving as a first-line anticounterfeiting measure. In emission mode, different ε_LWIR_ between the front and back side of the devices can be identified by an IR camera, serving as the second-line anticounterfeiting measure. In transmittance mode, temperature induced the achievement of luminous (Δ*T*_lum_), NIR (Δ*T*_IR_), and solar (Δ*T*_sol_) modulation that can only be recognized by experts, serving as the third-line anticounterfeiting measure. (D) Schematic diagram of the preparation process of the thermoresponsive photonic crystal hydrogel film (TRPCHF). (E) Schematic structure of the multimodal, multiband, and multiple anticounterfeiting device. (F) Schematic illustration of the mechanism of the color changing with temperature.

In this work, we propose a set of characteristics for a new generation of anticounterfeiting device that is capable of multimodal (reflection, transmission, and emission), multiband (visible, near-infrared [NIR], and long-wave infrared), and multiple anticounterfeiting with ultrahigh-temperature sensitivity (Fig. 1A to C). As for the first-line anticounterfeiting measure, in reflection mode, the lattice constant of the thermoresponsive photonic crystal hydrogel film (TRPCHF) varies with temperature, which leads to the observed structural colors and spectral changes (Fig. 1A). For the second-line anticounterfeiting measure, in emission mode, the device achieves a high long-wave infrared (LWIR, 2.5 to 25 μm) emissivity (ε_LWIR_) in the back side and a low ε_LWIR_ in the front side, which can be identified by an infrared (IR) camera (Fig. 1B). For the most important third-line anticounterfeiting measure, in transmittance mode, luminous (Δ*T*_lum_, 0.38 to 0.78 μm), NIR (Δ*T*_NIR_, 0.78 to 2.5 μm), and solar (Δ*T*_sol_, 0.38 to 2.5 μm) modulation can only be achieved by experts assisted with Vis–NIR spectra instruments by controlling the temperature (Fig. 1C). This proposed device herein is expected to achieve high-performance optical anticounterfeiting, and may have potential applications in smart windows, sensors, camouflage, and so on.

## Results and Discussion

Figure 1D and E schematically illustrate the fabrication process and structure of the proposed anticounterfeiting device. As schemed in Fig. 1D, a precursor solution consisting of ethylene glycol (EG, which served as a solvent), Fe_3_O_4_@poly(vinylpyrrolidone) colloidal nanocrystal clusters (Fe_3_O_4_@PVP CNCs, which served as an assembly unit), *N*-isopropylacrylamide (NIPAM, a thermo-responsive monomer), ethylene glycol dimethacrylate (EGDMA, which served as a cross-linker), and 2-hydroxy-2-methylpropiophenone (HMPP, which served as a photoinitiator) was injected into a narrow gap between 3-(trimethoxysilyl)propyl methacrylate (TMSPMA)-modified glass (Fig. S1) and glass. Under an external magnetic field (*H*), the Fe_3_O_4_@PVP CNCs were assembled into one-dimensional (1D) chain-like PC arrays, which were fixed in PNIPAM matrix after the ultraviolet (UV)-initiated in situ free radical polymerization. The resultant TRPCHF covalently anchoring onto the TMSPMA-modified glass is further exposed to water to remove the unreacted components and attain a state of swelling equilibrium. Subsequently, a 0.5-mm gasket and single-sided indium-tin oxide (ITO) glass are used to seal the TRPCHF to produce the final anticounterfeiting device (Fig. 1E). The observed structural colors of the device can be modulated by temperature changes (Fig. 1F), thereby serving as a first-line anticounterfeiting measure. The device possesses distinctive IR characteristics that can be identified through the use of an IR camera, representing a second-line anticounterfeiting measure. Of utmost importance, the luminous transmittance modulation (Δ*T*_lum_), the solar transmission modulation (Δ*T*_sol_), and the IR transmission modulation (Δ*T*_IR_) can only be identified by experts through temperature manipulation and subsequent calculation, constituting a third-line anticounterfeiting measure. The device displays a multimodal response effect, featuring reflection, emission, and transmission, across multiband (Vis–NIR–LWIR), which offers a new way for high-level anticounterfeiting applications.

**Fig. 2.**
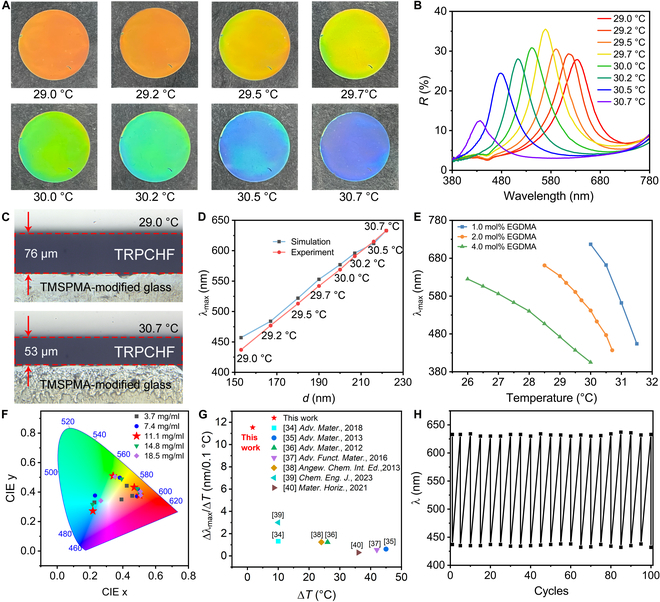
Characterization of the TRPCHF. (A) Digital photographs and (B) corresponding reflection spectra of the TRPCHF fabricating by 2.0 mol% EGDMA at different temperatures. (C) Side view of the TRPCHF at 29 and 30.7 °C. (D) λ_max_ as a function of interparticle distances. (E) λ_max_ of TRPCHF prepared with different content of EGDMA as a function of water temperature. (F) Corresponding Commission International de L’Eclairage (CIE) chromaticity diagram of Figure S9. (G) Sensitivity contrast of the previous thermochromic materials based on PC structure (the related references were Refs. [37–43]). (H) Cycling performance.

Figure S2 presents the cross-section scanning electron microscopy (SEM) images of the TRPCHF prepared with 2.0 mol% EGDMA. These images reveal the formation of 1D chain-like PC structures composed of Fe_3_O_4_@PVP CNC particles, which are effectively immobilized within the PNIPAM networks. The observed particle chains, characterized by regular interparticle spacing, leads to the manifestation of structural color through Bragg diffraction phenomena. In Fig. 2A, digital photographs of the TRPCHF are presented under varying temperature conditions. Correspondingly, Fig. 2B depicts the reflection spectra associated with these temperature-dependent images. According to the Bragg diffraction equation:

$$\lambda_{\max}=2\mathrm{nd}\;\text{sin}\;\theta$$ (1)

where *λ*_max_ is the diffracted wavelength, *n* represents the effective refractive index, *d* denotes the center-to-center distance of 2 adjacent Fe_3_O_4_@PVP nanoparticles (interparticle distances), and *θ* refers to the angle between the incident light and diffraction plane. As the temperature gradually increased from 29 to 30.7 °C, the TRPCHF underwent a noticeable color change from red to blue, causing the *λ*_max_ to shift from 633 to 437 nm (Fig. 2B). The optical microscope image of the cross-section of the device is shown in Fig. 2C and the thickness of the TRPCHF under different temperature conditions could be obtained. At 29 °C, the thickness was measured to be 76 μm, whereas at 30.7 °C, it reduced to 53 μm. This resulted in a calculated thickness ratio of 1.43, which closely aligns with the blueshift ratio of 1.45 observed in the corresponding diffraction peaks. This correlation reinforces the notion that the observed color change is attributed to the variations in the interparticle distances (*d*) due to gel shrinkage induced by temperature fluctuations. Finite-difference time-domain (FDTD) simulations are conducted to understand the relationship between *d* and *λ*_max_ (Fig. 2D), in accordance with the experimental results.

It is worth noting that the cross-linking of PNIPAM polymers plays a pivotal role in inducing shrinkage, which, in turn, influences the thermochromic properties of the PC films (Figs. S2 to S4). Digital photographs and thermochromic properties of the TRPCHF fabricated using varying EGDMA concentrations are depicted in Figs. S5 to S7. Figure 2E summarizes the *λ*_max_ of the TRPCHF as a function of water temperature. The obtained TRPCHF exhibits a blue-shifted color at the initial temperature (26.5 °C) with increasing EGDMA content, and this phenomenon can be attributed to the contraction of the PNIPAM matrix, which reduces *d* as the polymer cross-linking increases. Although the TRPCHF with 0.5 mol% EGDMA displays the highest sensitivity, the full width at half maximum (FWHM) is broad, which means a relatively low saturation (Fig. S5). This may be attributed to the significant expansion of the polymer during the water washing process, resulting in partial destruction of the ordered structure. In contrast, the TRPCHF prepared with 4.0 mol% EGDMA exhibits increased color saturation but decreased sensitivity, as observed in Fig. 2E and Fig. S7. These observations indicate that an increase in cross-linking degree limits the expansion and contraction of the polymer chain, thereby reducing the wavelength tuning range.

The optical properties of TRPCHF are also affected by both the concentration of Fe_3_O_4_@PVP CNCs and the thickness of the films. The adhesion of the TRPCHF to the TMSPMA-modified glass substrate may be compromised if the concentration of Fe_3_O_4_@PVP CNCs in the precursor solution is larger than 18.5 mg/ml and the gasket is thicker than 200 μm. This phenomenon may be attributed to the strong light absorption of Fe_3_O_4_, which limits the penetration depth of UV light, thus resulting in poor bonding between the TRPCHF and the glass substrate and the formation of cracks after swelling equilibrium in water (Fig. S8). Furthermore, the pattern behind were blurred when viewed through them, meaning that the transparency of the prepared TRPCHF can decrease with increasing concentration and thickness of the film, as evidenced by the data presented in Fig. S8.

The reflection spectra (Fig. S9) and the corresponding chromaticity diagram in the CIE color space (Fig. 2F) demonstrate that optimal concentration for the fabrication of TRPCHF is 11.1 mg/ml of Fe_3_O_4_@PVP CNCs, as the point is in close proximity to the CIE horseshoe rim at all temperatures, suggesting a relatively pure color. Figures S10 and S11 present digital photographs and their corresponding reflection spectra of TRPCHF prepared with thicknesses of 100 and 200 μm at various water temperatures, respectively. The reduction in size of responsive PCs can enhance the response time considerably [35,36]. Furthermore, by taking into account the effect of gel thickness on color saturation (Fig. 2B and Figs. S10 and S11), a thickness of 50 μm was identified as the optimized condition.

The strength of the external *H* is a crucial parameter to fabricate TRPCHF. Inadequate strength of *H*, such as 200 Gs, results in a low ordering of the chain-like structure obtained in the precursor solution, leading low spectral reflectivity and broad FWHM, as demonstrated in Fig. S12. On the other hand, increasing the strength of *H* during the polymerization process leads to a significant blueshift in *λ*_max_, as shown in Fig. S13, giving TRPCHF with narrow FWHM (Fig. 2B).

The temperature sensitivity of the TRPCHF exceeds that of all previously reported thermochromic materials based on PC structures (Fig. 2G). Such high sensitivity is attributed not only to the excellent thermoresponsive performance of PNIPAM but also to the covalent anchoring of PNIPAM to TMSPMA-modified glass substrates. The presence of covalent bonds between the PNIPAM chains and the TMSPMA-modified glass, along with the cross-linked structure of the TRPCHF, enhanced the adhesive strength between the TRPCHF and the TMSPMA-modified glass. As the temperature increases, the adhesion between the TRPCHF and the TMSPMA-modified glass limits the shrinkage in the horizontal direction (Fig. 2C), which is in contrast to the TRPCHF prepared without adhesive (Fig. S14). This isotropic shrinkage behavior results in a more pronounced reduction of the lattice constant, which is responsible for the high temperature sensitivity observed in the TRPCHF. In practical applications, cycling durability is a critical requirement. Movie S1 describes that the temperature-responsive color change is reversible. Additionally, the durability test of the 50-μm TRPCHF sample was conducted over 100 cycles between 29 and 30.7 °C, indicating relatively constant *λ*_max_ during thermal cycling (Fig. 2H).

**Fig. 3.**
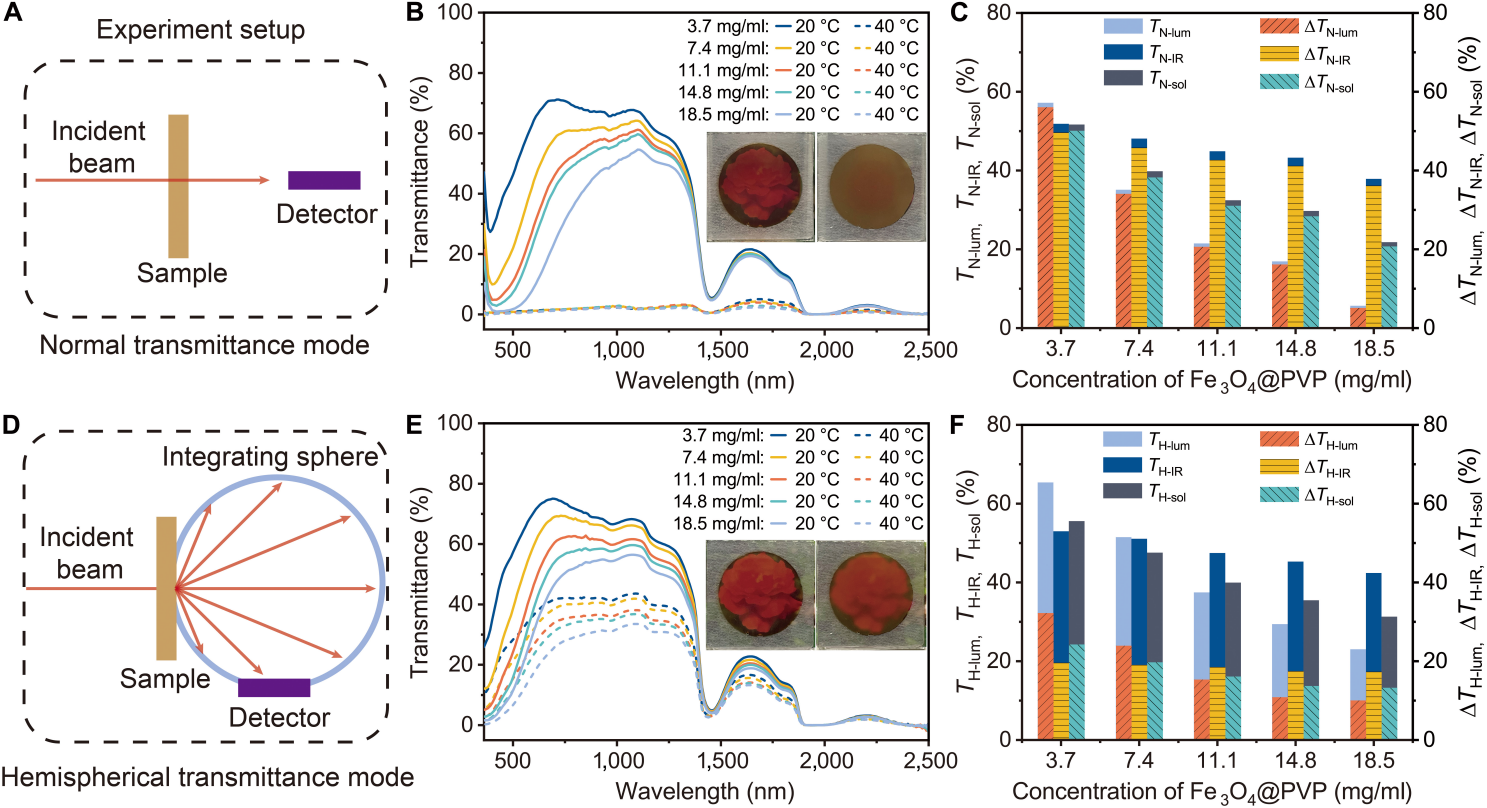
Optical transmittance. (A) Illustration of the spectrum measurement setup of normal transmittance (*T*_N_). (B) Normal transmittance spectra for the devices at 20 °C (solid line) and 40 °C (dashed line) prepared with different concentrations of Fe_3_O_4_@PVP CNC particles, and (C) the corresponding comparison of optical performance. (D) Illustration of the spectrum measurement setup of hemispherical transmittance (*T*_H_). (E) Normal transmittance spectra for the devices at 20 °C (solid line) and 40 °C (dashed line) prepared with different concentrations of Fe_3_O_4_@PVP CNC particles, and (F) the corresponding comparison of optical performance.

To comprehensively investigate the influence of temperature on the optical properties of the sample in transmission mode, a rigorous experimental analysis was performed. In the normal transmittance mode (Fig. 3A), the measurement setup captures only the beam passing through the samples aligned with the incident direction. As shown in Fig. 3B, at a temperature of 20 °C (below LCST), the normal luminous transmittance (*T*_N-lum_) is 57.2% for samples prepared with 3.7 mg/ml of Fe_3_O_4_@PVP CNCs. However, with an increase in the concentration of Fe_3_O_4_@PVP CNCs to 18.5 mg/ml, the *T*_N-lum_ decreases to 5.7%. This trend is also evident in the optical photos (Fig. S15), indicating that the strong absorption of visible light by Fe_3_O_4_@PVP CNC is the main cause for this change. As the temperature rises to 40 °C (above the LCST), the phase transition of PNIPAM triggers the shrinkage of the polymeric chain, resulting in the formation of scattering centers and a decrease in *T*_N-lum_ for all samples (Fig. S15). The calculated optical properties are shown in Fig. 3C, revealing that the modulation of normal luminous transmittance (Δ*T*_N-lum_), IR transmittance (Δ*T*_N-IR_), and solar transmittance (Δ*T*_N-sol_) gradually decreases with an increase in the concentration of Fe_3_O_4_@PVP CNCs. For instance, the calculation indicates that Δ*T*_N-lum_ decreases from 56.0% to 5.1% as the concentration of Fe_3_O_4_@PVP CNCs increases from 3.7 to 18.5 mg/ml. Similarly, Δ*T*_N-sol_ decreases from 50.1% to 20.7%, and Δ*T*_N-IR_ decreases from 49.6% to 36.1%. It is noteworthy that the significant change in *T*_N-IR_ is not detectable by the human eye and can only be identified by instruments, which makes it conducive to anticounterfeiting purposes.

In hemispherical transmittance mode (Fig. 3D), the experimental setup involves the use of an integrating sphere to capture the entirety of light transmission through the sample. Comparative analysis with the normal transmittance mode reveals that all samples exhibit higher luminous transmittance and IR transmittance at 20 °C, as depicted in Fig. 3E. For instance, as the concentration of Fe_3_O_4_@PVP CNC particles in the fabrication process increases from 3.7 to 18.5 mg/ml, the hemispherical luminous transmittance (*T*_H-lum_) decreases from 65.4% to 23.0%, which is further supported by optical photos in Fig. S16. Additionally, the hemispherical IR transmittance (*T*_H-IR_) decreases from 53.0% to 42.4%. Analysis of the calculated optical properties in Fig. 3F reveals that the modulation of hemispherical luminous transmittance (Δ*T*_H-lum_), IR (Δ*T*_H-IR_), and solar (Δ*T*_H-sol_) also gradually decreases with the increase of Fe_3_O_4_@PVP CNC concentration, though the magnitude of the decrease varies significantly. Specifically, Δ*T*_H-lum_ decreases from 32.2% to 10.0%, while Δ*T*_H-IR_ only changes from 19.6% to 17.3%. Furthermore, we have also conducted evaluations of the optical performance and modulation ability of samples prepared with varying amounts of cross-linking agents at different temperatures, as depicted in Figs. S17 and S18.

**Fig. 4.**
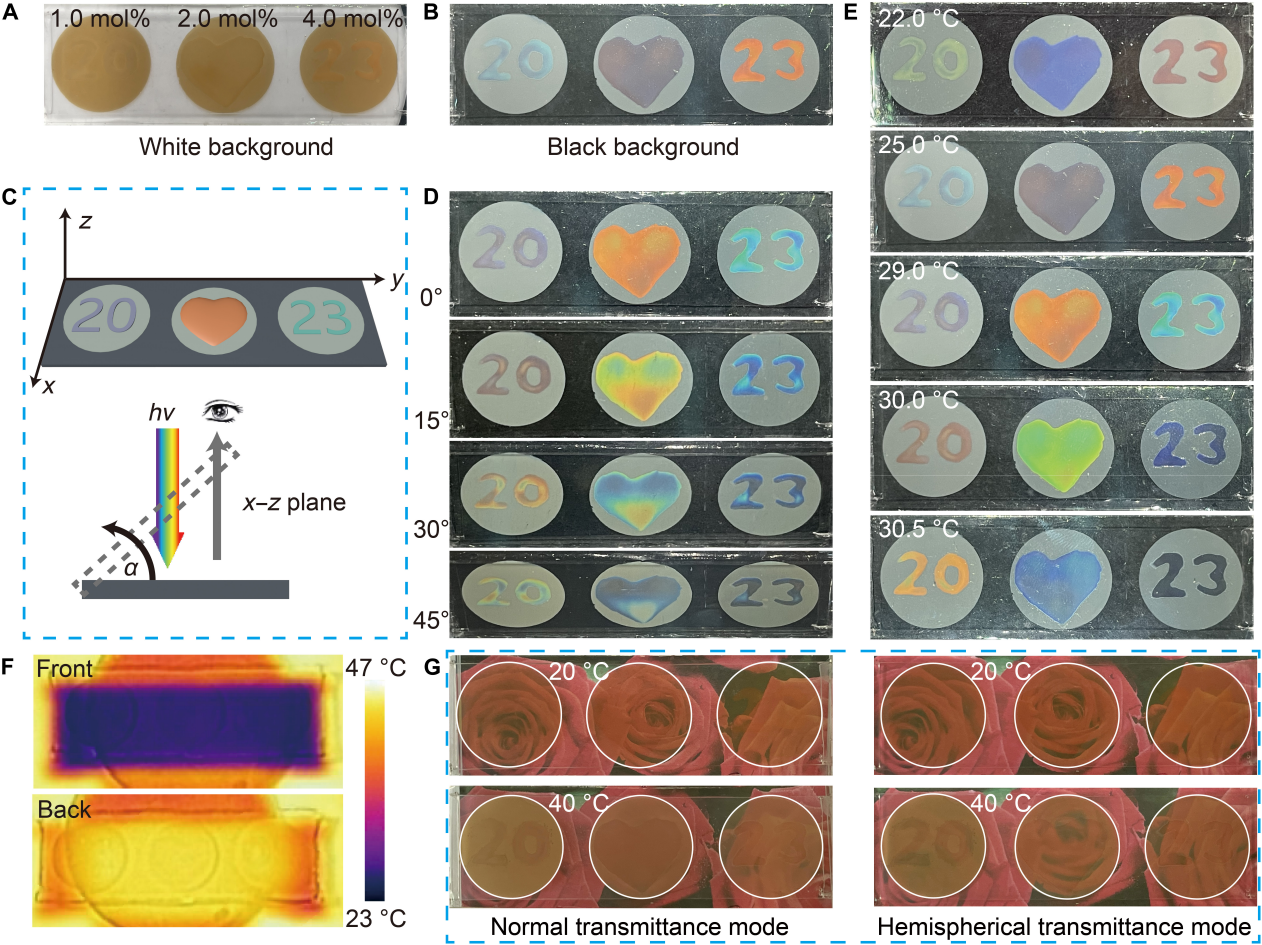
The application of multimodal, multiband, and multiple anticounterfeiting devices. Digital photographs of the device placed on the (A) white and (B) black background. (C) The definition of tilting angle and (D) the taken corresponding digital photos. (E) Digital photographs of the device under different temperatures. (F) IR images for the low-E side (front) and high-E side (back) of the device at 40 °C, respectively. (G) The digital photographs of the device in the normal and hemispherical transmittance mode.

To demonstrate the multiple anticounterfeiting applications, different cross-linking agents 1.0, 2.0, and 4.0 mol% were used in the fabrication of patterns “20”, “❤”, and “23”, respectively (Fig. S19). On a white background, the appearance of the device is brownish (Fig. 4A). Interestingly, when placed on a black background, the structural color of the pattern became clearly visible due to the effective suppression of incoherent scattering (Fig. 4B). At 29 °C, when the device was tilted from 0° to 45°, the pattern was easily recognizable, and the color blue shifted (Fig. 4C and D). Figure 4E shows that the pattern displayed different colors at different temperatures due to the diverse cross-linking degrees and thermoresponsive. Consequently, this color transformation serves as a first-line anticounterfeiting measure. Owing to the distinct materials employed on either side of the device, the LWIR emissivity (*ε*_LWIR_) differs between 2 sides (Fig. S20). IR images were captured for the front and back sides of the device at a temperature of 40 °C. The front side of the device appears blue in the IR photograph due to its considerably low *ε*_LWIR_, as the ITO coating emits less IR than the background and appears darker (Fig. 4F). Conversely, the back side of the device displays a white color, as it possesses a higher *ε*_LWIR_ (*ε*_LWIR_ = 0.89) than the background, and hence emits more LWIR than the background, thereby appearing brighter under the IR camera. These results can serve as a second-line anticounterfeiting measure. Figure 4G shows the digital photographs of the device in the normal and hemispherical transmittance modes. It reveals that the thermochromic attributes of PNIPAM enables the devices to implement transmittance adjustment via temperature stimuli, permitting the calculation of Δ*T*_lum_ and Δ*T*_sol_ through complex formulas, thereby yielding a third-line anticounterfeiting measure (Fig. 3C and F). The above analysis indicates that the device can achieve multiple anticounterfeiting effects with multimode and multiband variation and high temperature sensitivity.

## Conclusion

In summary, we have demonstrated a simple strategy to design and develop the multimodal, multiband, and multiple anticounterfeiting device with high temperature sensitivity. In this device, a PNIPAM-based TRPCHF was chemically anchored by a TMSPMA-modified glass and then covered with ITO glass. The device could achieve multimodal effect in transmittance mode, reflection mode, and emission mode by integrating optical transmittance, structural color, and IR, which can perform first-line and second-line anticounterfeiting tasks. A record high temperature sensitivity of up to 11.5 nm/0.1 °C was demonstrated due to the blueshift in peak diffraction wavelength in the visible range. Importantly, the device holds a third-line anticounterfeiting feature, which only expert can identify by quantifying the transmission modulation through the temperature change. This new-generation device demonstrated a conceptual advancement in high-performance optical anticounterfeiting applications and could be leveraged in other optical modulation applications such as smart windows, sensors, and camouflage.

## Materials and Methods

### Materials

NIPAM (≥99%, Product No. 731129), TMSPMA (≥97%, Product No. M6514), EGDMA (98%, Product No. 335681), HMPP (97%, Product No. 405655), EG (≥99%, Product No. 102466), polyvinylpyrrolidone (PVP, K30, Product No. 81420), tannic acid (Product No. 403040), sodium acetate (≥99%, Product No. S8750), and iron(III) chloride hexahydrate (FeCl_3_·6H_2_O, 97%, Product No. 236489) were used in the study. All chemicals were used as received without further purification. Super-purity water (18.20 MΩ cm) was produced by the milli-Q system (Millipore, USA). The glass and single-side ITO glass with a thickness of 1.1 mm were purchased from Winteck Technology.

### Preparation of the TMSPMA-modified glass

The clean glass was immersed in a “piranha” solution consisting of concentrated sulfuric acid (98 wt.%) and hydrogen peroxide solution (30 wt.%) in a volumetric ratio of 7:3 and then boiled for 1 h. Subsequently, the glass was rinsed with ethanol and water, respectively, and dried under nitrogen gas to obtain the hydrophilic glass. Next, the hydrophilic glass was immersed in a solution composed of 30 ml of toluene and 30 μl of TMSPMA for 24 h, after which it was washed thoroughly with ethanol and dried with nitrogen to obtain the TMSPMA-modified glass.

### Preparation of the thermal-responsive photonic crystal films

In a typical preparation process, NIPAM (0.2263 g, 2 mmol), EGDMA (0.04 mmol), HMPP (0.04 mmol), and Fe_3_O_4_@PVP CNCs (6 mg) were dissolved into 0.3395 g of EG to form a homogeneous precursor solution (0.54 ml) via intense sonication. Then, 30 μl of the precursor solution was injected into a gap (50 μm) formed by a gasket (2 cm in inside diameter) sandwiched between the TMSPMA-modified glass and the clean glass. After that, it was placed above a round NdFeB permanent magnet (Φ10 × 1 cm) providing a magnetic field strength of 500 Gs for 1 min. In such a way, the superparamagnetic Fe_3_O_4_@PVP CNCs in the solution were self-assembled into 1D chain-like PC arrays, resulting in a bright iridescence before polymerization. After exerting the UV light (UVEC-4II, 365 nm, 40 mW cm^−2^) for 300 s, the TRPCHF adhering on the TMSPMA-modified glass was obtained. The film was sufficiently swollen to equilibrium in water at 22 °C. A series of TRPCHFs were prepared by changing parameters such as the concentration of Fe_3_O_4_@PVP CNCs, EGDMA, and the intensity of magnetic field.

### Preparation of the multimodal, multiband, and multiple anticounterfeiting devices

The sandwiched device was loaded with the precursor solution, and the black photomask “20❤23” pattern was placed onto the upper glass plate. Subsequently, UV light was applied for 2 min to the device, followed by removing the black mask pattern from the device. Further polymerization occurred for 3 min under UV light and a magnetic field strength of 500 Gs. The content of the cross-linking agent of “20”, “❤”, and “23” is 1.0, 2.0, and 4.0 mol%, respectively. After washing with water and reaching equilibrium swelling in water at 22 °C, a 1-mm 3M tape was used to create a gap, and then a single-sided ITO glass was covered.

### Characterization

All the digital photos were taken by Apple iPhone 12 Pro. Field-emission SEM images were conducted on a Carl Zeiss Supra 55 scanning electron microscope. The reflection spectra were measured with a fiber-optic spectrometer (Avantes AvaSpec-ULS2048L Starline), with temperature control achieved through a water bath apparatus monitored by a mercury thermometer. For the hemispherical transmittance test, the spectra were measured by the UV–Vis–NIR spectrophotometry (Lambda 950, Perkin Elmer) equipment with 150-mm integrating spheres for signal collection. For the normal transmittance test, the UV–Vis–NIR spectra for the sample were measured with the UV–Vis–NIR spectrophotometer system (Avantes AvaSpec-ULS2048L Starline Versatile Fiber-optic Spectrometer and AvaSpec-NIR256-2.5-HSC-EVO). Both of the spectrophotometer was equipped with a heating and cooling stage (Linkam PE120) to control the sample temperature. Prior to each measurement, samples were placed on the stage and allowed to thermally equilibrate for at least 10 min to ensure temperature stabilization before spectral acquisition.

The transmittance *T*_lum_, *T*_TR_, and *T*_sol_ were calculated using the equation:

$$T_{\mathrm{lum}/\mathrm{IR}/\mathrm{sol}}=\int \varphi_{\mathrm{lum}/\mathrm{IR}/\mathrm{sol}}T\left(\lambda \right)\mathrm{d\lambda}/\int \varphi_{\mathrm{lum}/\mathrm{IR}/\mathrm{sol}}\mathrm{d\lambda}$$ (2)

where *T*(λ) is the spectral transmittance (360 to 780 nm for *T*_lum_, 790 to 2,300 nm for *T*_IR_, and 360 to 2,300 nm for *T*_sol_). *φ*_lum_(λ) is the standard luminous efficiency function of photopic vision for the wavelength of 380 to 780 nm while *φ*_IR_(λ) and *φ*_sol_(λ) are the IR/solar irradiance spectra for air mass 1.5 (corresponding to the sun standing 37° above the horizon with 1.5 atmosphere thickness, which corresponds to a solar zenith angle of 48.2°), respectively.

$$∆T_{\mathrm{lum}/\mathrm{IR}/\mathrm{sol}}=T_{\mathrm{lum}/\mathrm{IR}/\mathrm{sol},20{}^{\circ}C}-T_{\mathrm{lum}/\mathrm{IR}/\mathrm{sol},40{}^{\circ}C}$$ (3)

### Numerical simulation

The PC consisted of periodic layers of nanoparticles in a hydrogel medium. The calculation domain is a 3D domain. The layers of nanoparticle are 30. The radius of the nanoparticle is 75 nm. The distance between 2 nanoparticles is set to some certain value, which varies from 153 to 222 nm. The nanospheres were uniformly distributed in the hydrogel substrate along the *Z*-axis direction. The thickness of the hydrogel can just warp the nanoparticle’s array. Therefore, the dimension of the calculation domain in the *Z* axis is set to be larger than the thickness of the hydrogel, and the dimension of the calculation domain in the *X* and *Y* axis is set to 400 nm. The boundary condition is set to PML for all the 6 boundaries.

## Data Availability

All the data are included within the article and its supplementary material.
