# Supplementary material for: Multimodal, Multiband, and Multiple Anticounterfeiting Devices with Angle-Dependent Structural Color Highly Sensitive to Temperature
Source: Research (Wash D C). 2025 Oct 24;8:0919. doi: 10.34133/research.0919 (PMC12550300; doi:10.34133/research.0919)
Supplement: Supplementary 1 — Figs. S1 to S20 Movie S1 [file research.0919.f1.zip › Supporting Information.docx]

Supporting Information

**Multimodal, multiband, and multiple Anticounterfeiting Devices with Angle-depending Structural Color Highly sensitive to Temperature**

Gang Li, Shancheng Wang, Huaxu Liang, Wei Luo, Huiru Ma, Yi Long*, Jianguo Guan*

**Supporting Figures**


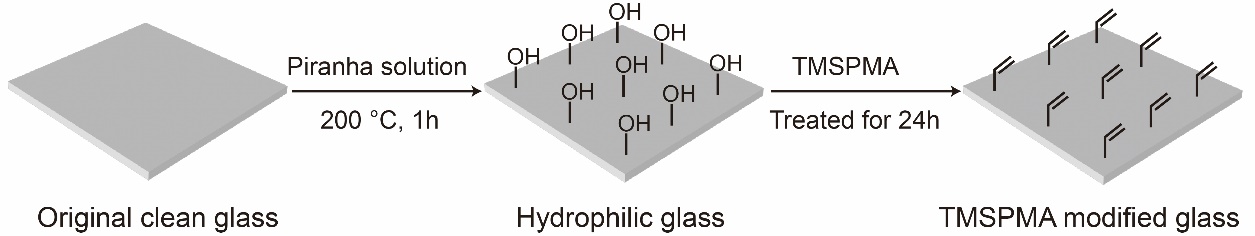


Figure S1. Schematic illustration of the process of the glass modified


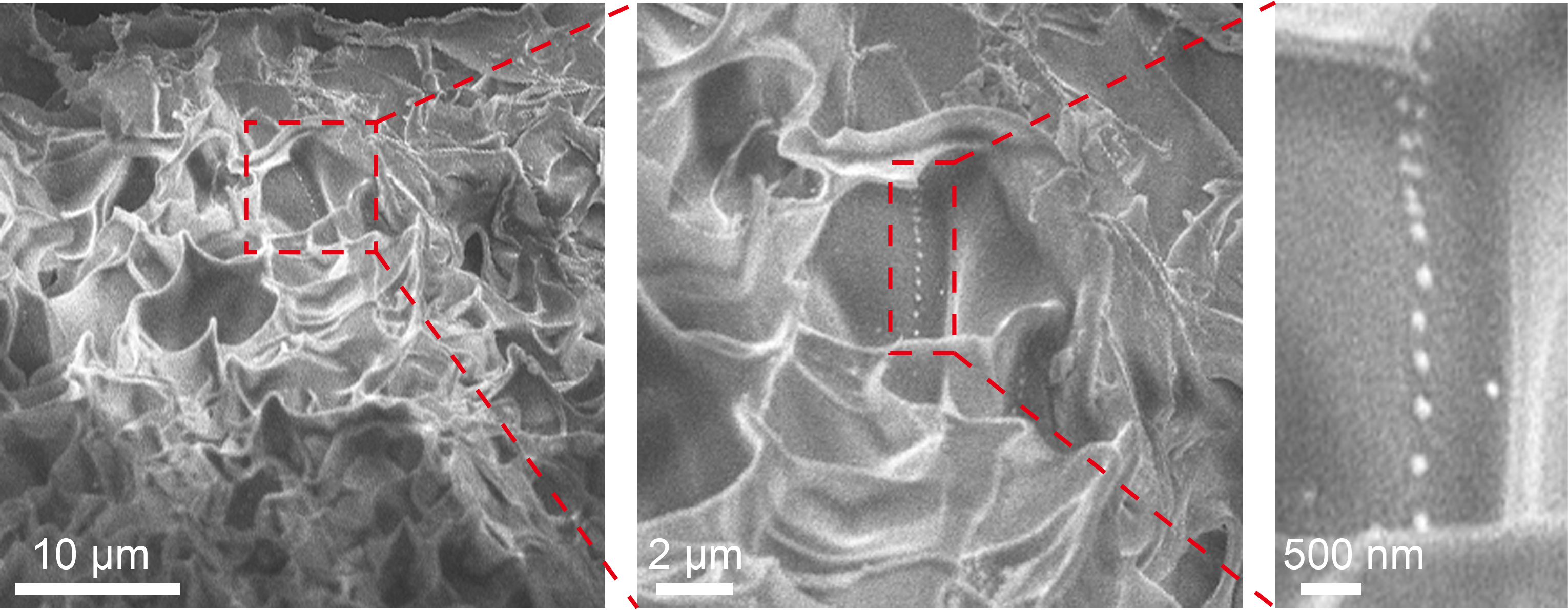


Figure S2. The cross-section SEM images of the TRPCHF preparing by 2.0 mol% EGDMA


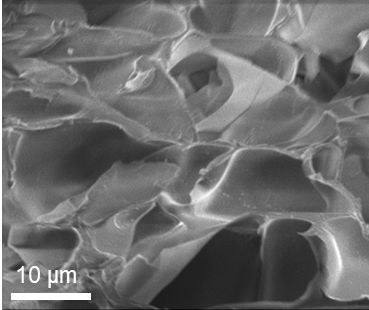


Figure S3. SEM image of the TRPCHF preparing by 1.0 mol% EGDMA


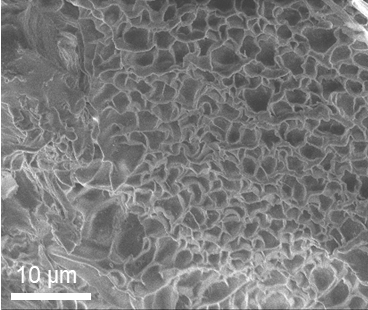


Figure S4. SEM image of the TRPCHF preparing by 4.0 mol% EGDMA


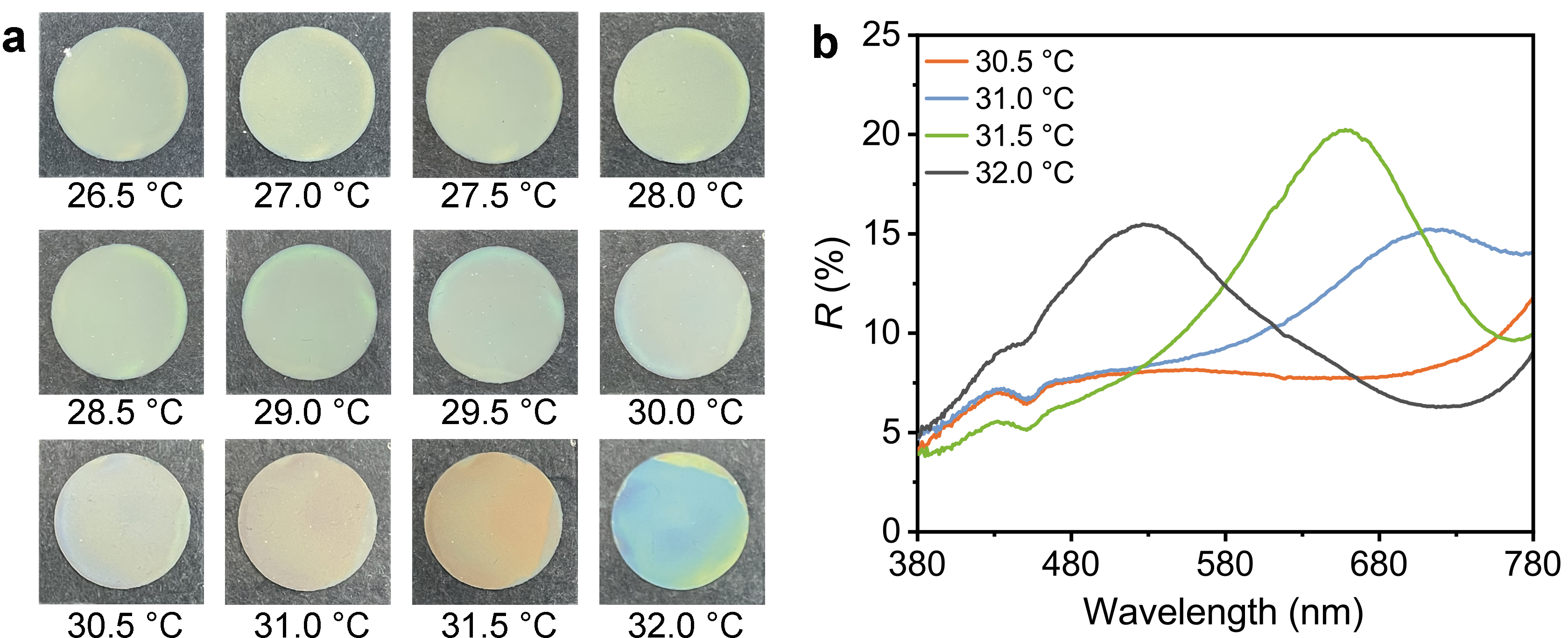


Figure S5. Digital photographs and corresponding reflection spectra of a 1D thermoresponsive photonic crystal film (TRPCHF) under different water temperatures. The TRPCHF was prepared by 0.5 mol% EGDMA.


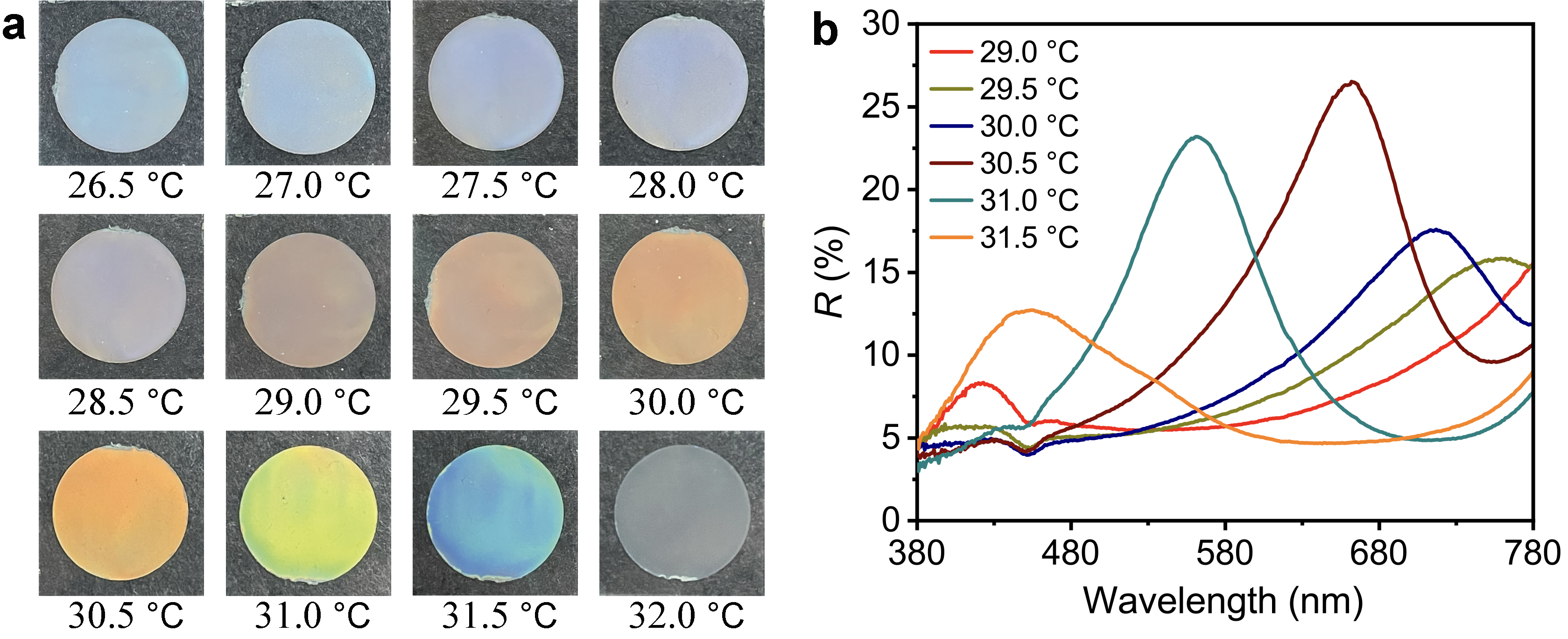


Figure S6. Digital photographs and corresponding reflection spectra of a 1D thermoresponsive photonic crystal film (TRPCHF) under different water temperatures. The TRPCHF was prepared by 1.0 mol% EGDMA.


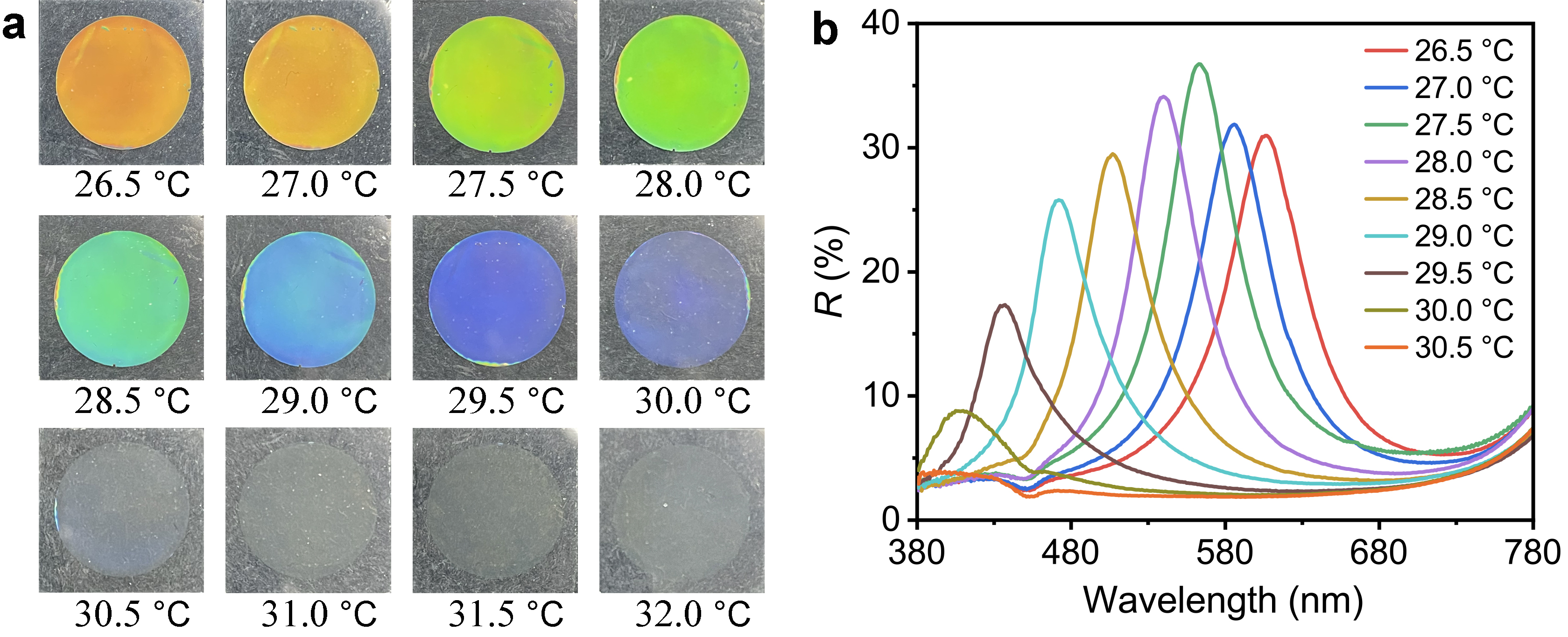


Figure S7. Digital photographs and corresponding reflection spectra of a 1D thermoresponsive photonic crystal film (TRPCHF) under different water temperatures. The TRPCHF was prepared by 4.0 mol% EGDMA.


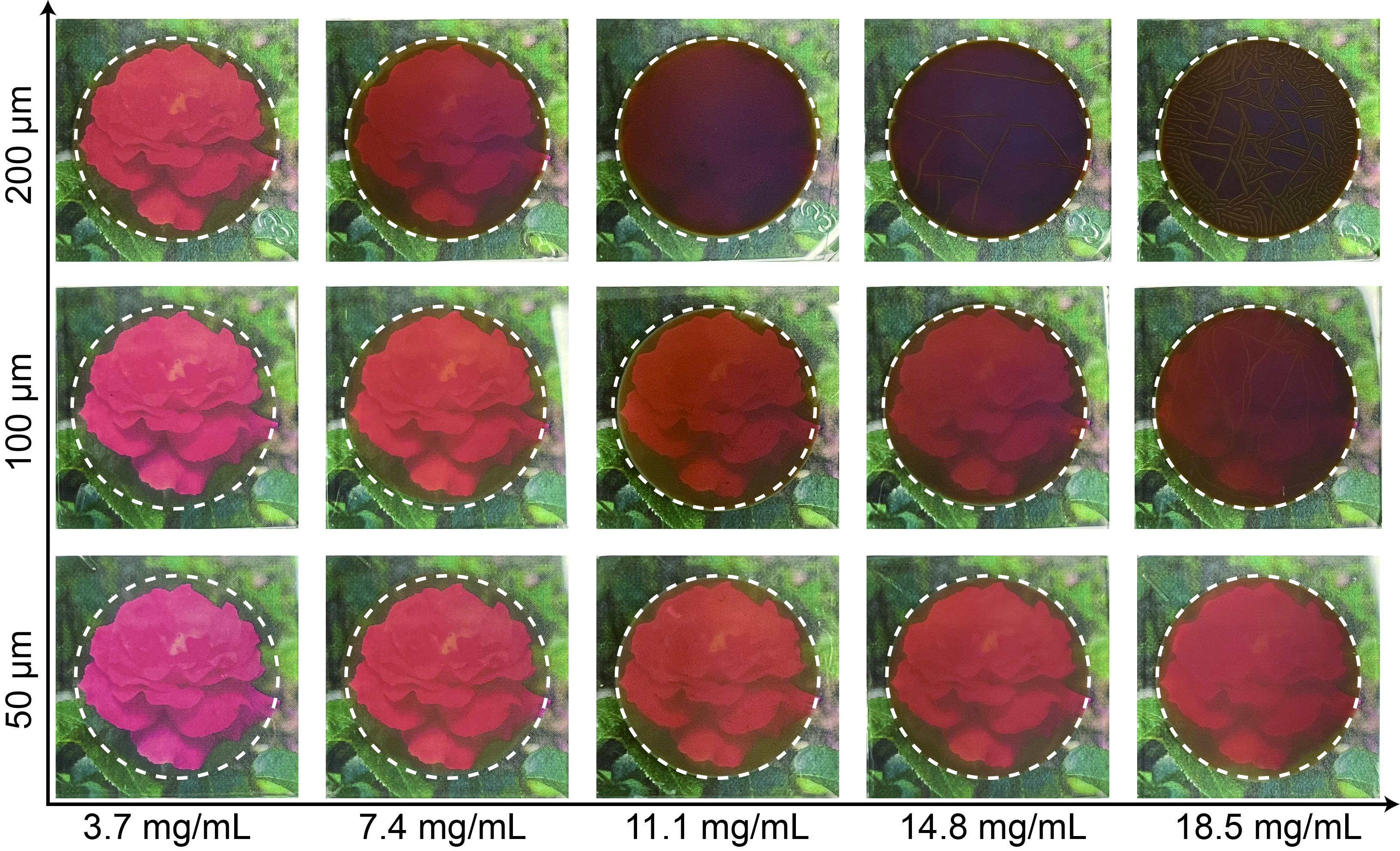


Figure S8. Digital photographs of TRPCHF preparing by different concentration of Fe_3_O_4_@PVP CNC particles and different thickness.


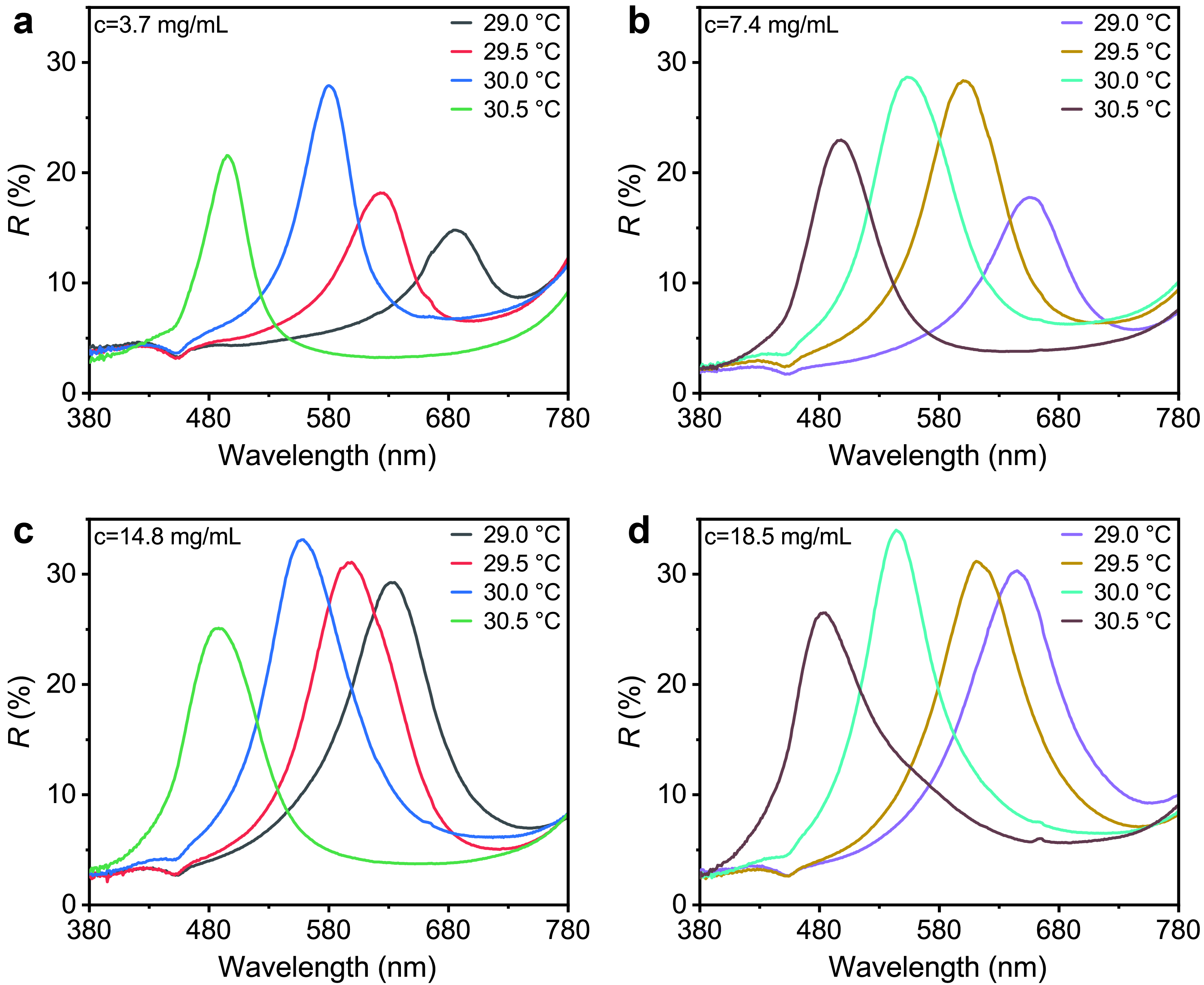


Figure S9. Reflection spectra of TRPCHF prepared by different concentration of Fe_3_O_4_@PVP CNC particles


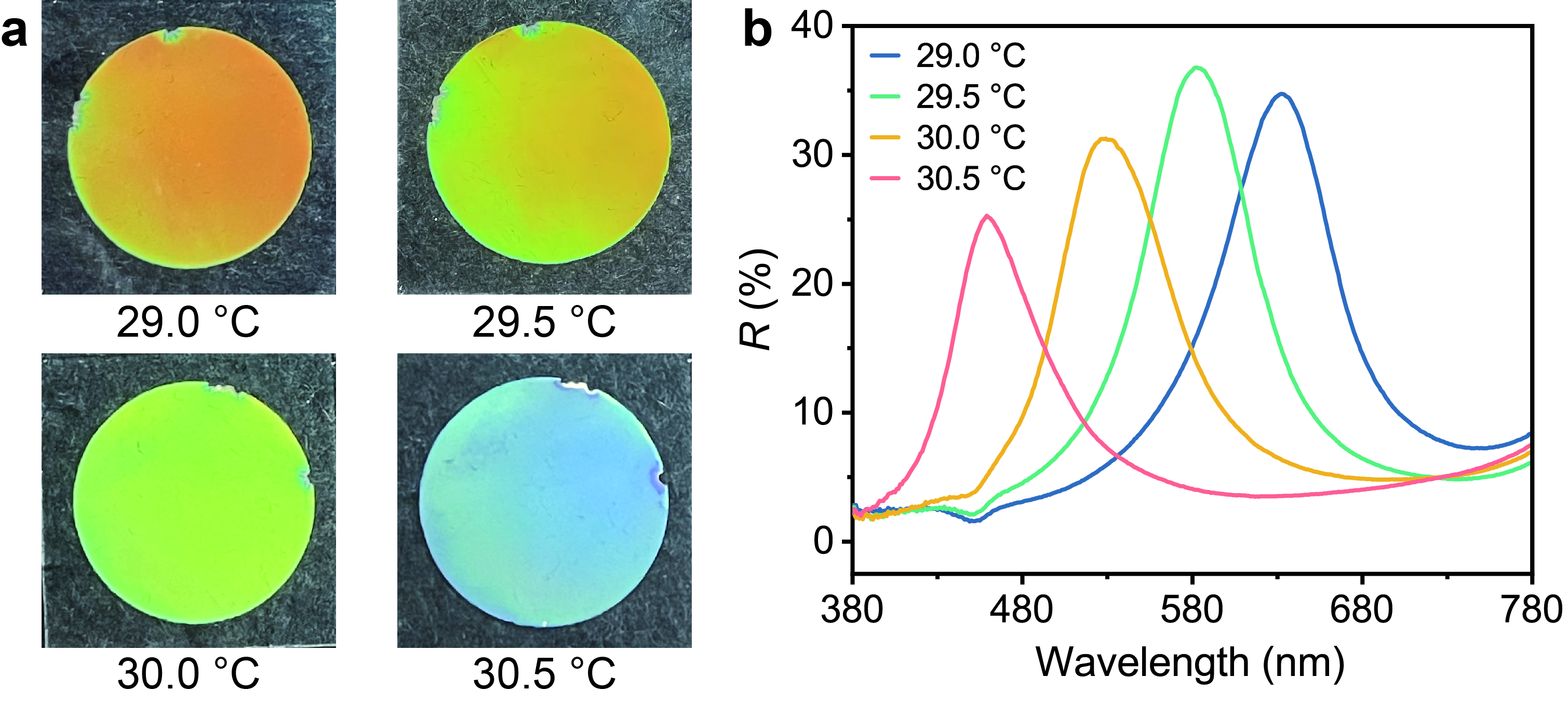


Figure S10. Digital photographs and corresponding reflection spectra of a 1D thermoresponsive photonic crystal film (TRPCHF) under different water temperatures. The TRPCHF was 100 μm.


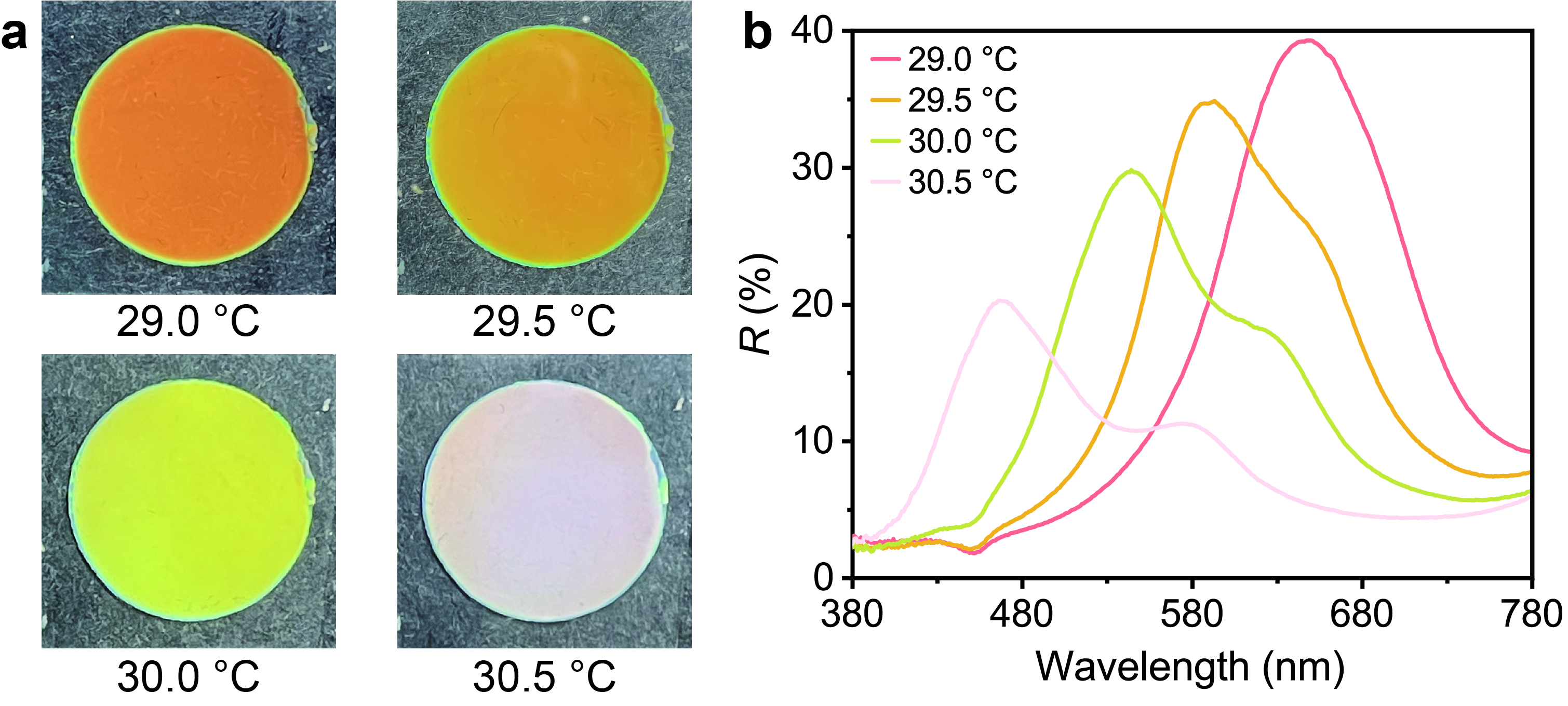


Figure S11. Digital photographs and corresponding reflection spectra of a 1D thermoresponsive photonic crystal film (TRPCHF) under different water temperatures. The thickness of TRPCHF was 200 μm.


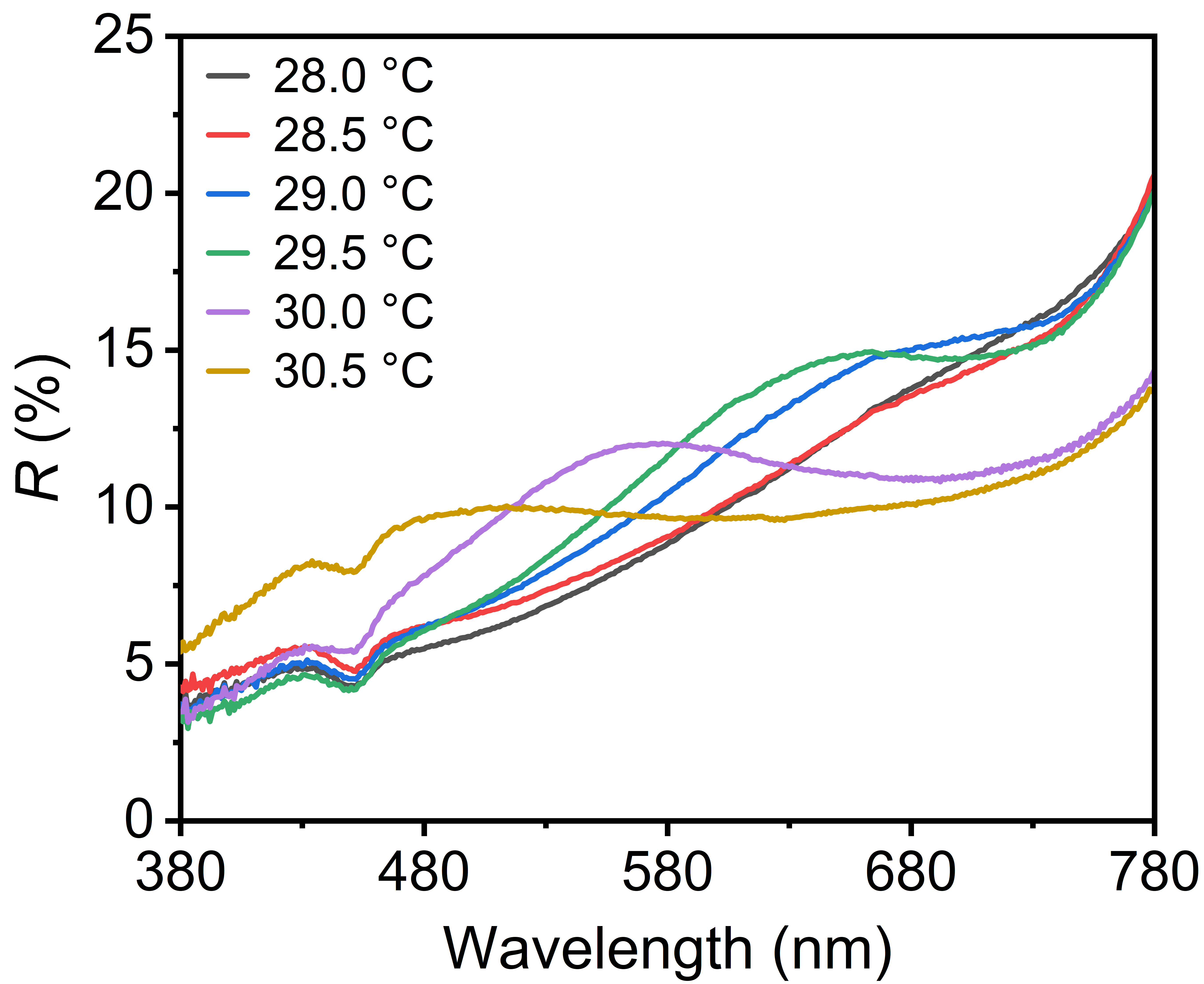


Figure S12. Digital photographs and corresponding reflection spectra of a 1D TRPCHF (prepared by 200 Gs) under different water temperatures.


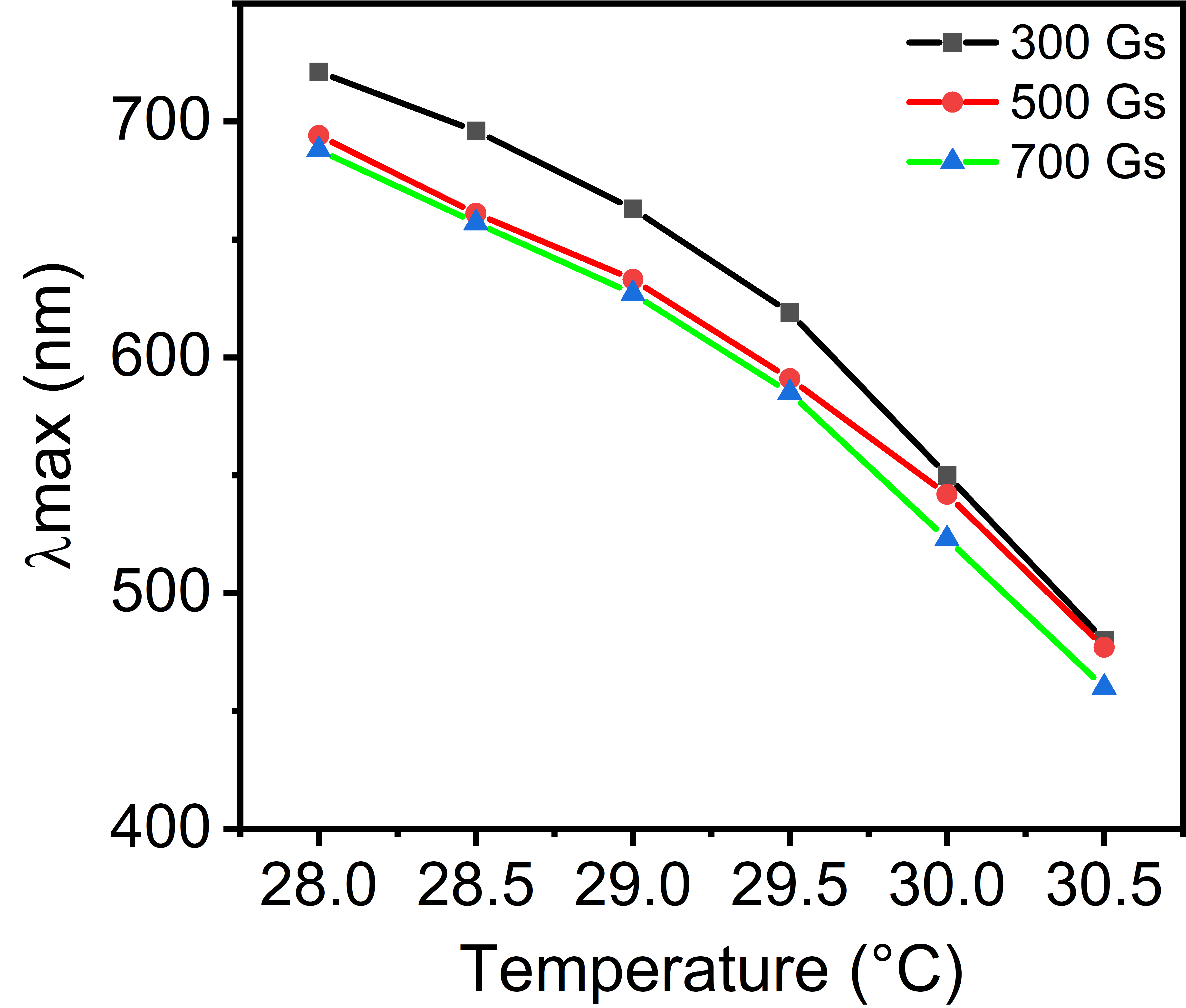


Figure S13. λ_max_ of TRPCHF preparing with different magnetic field strength as a function of water temperature.


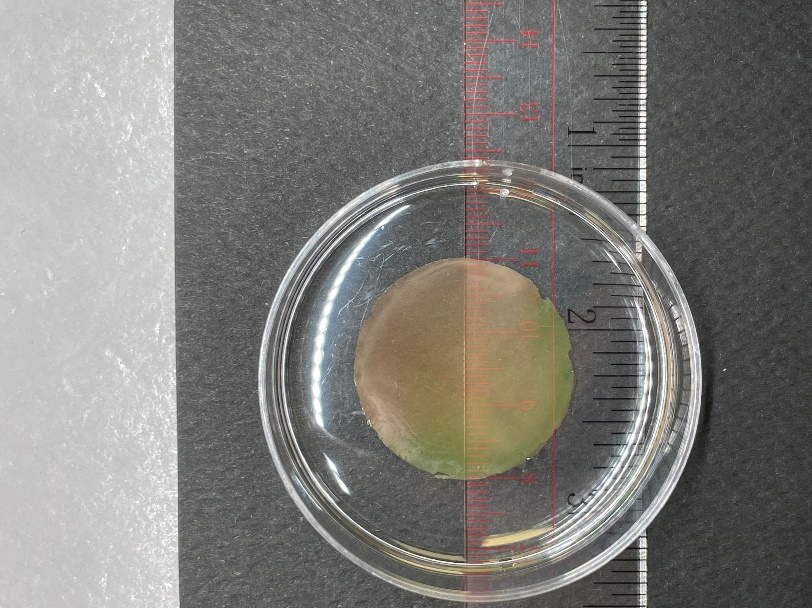


Figure S14. Digital photographs the 1D TRPCHF without adhesive the glass.


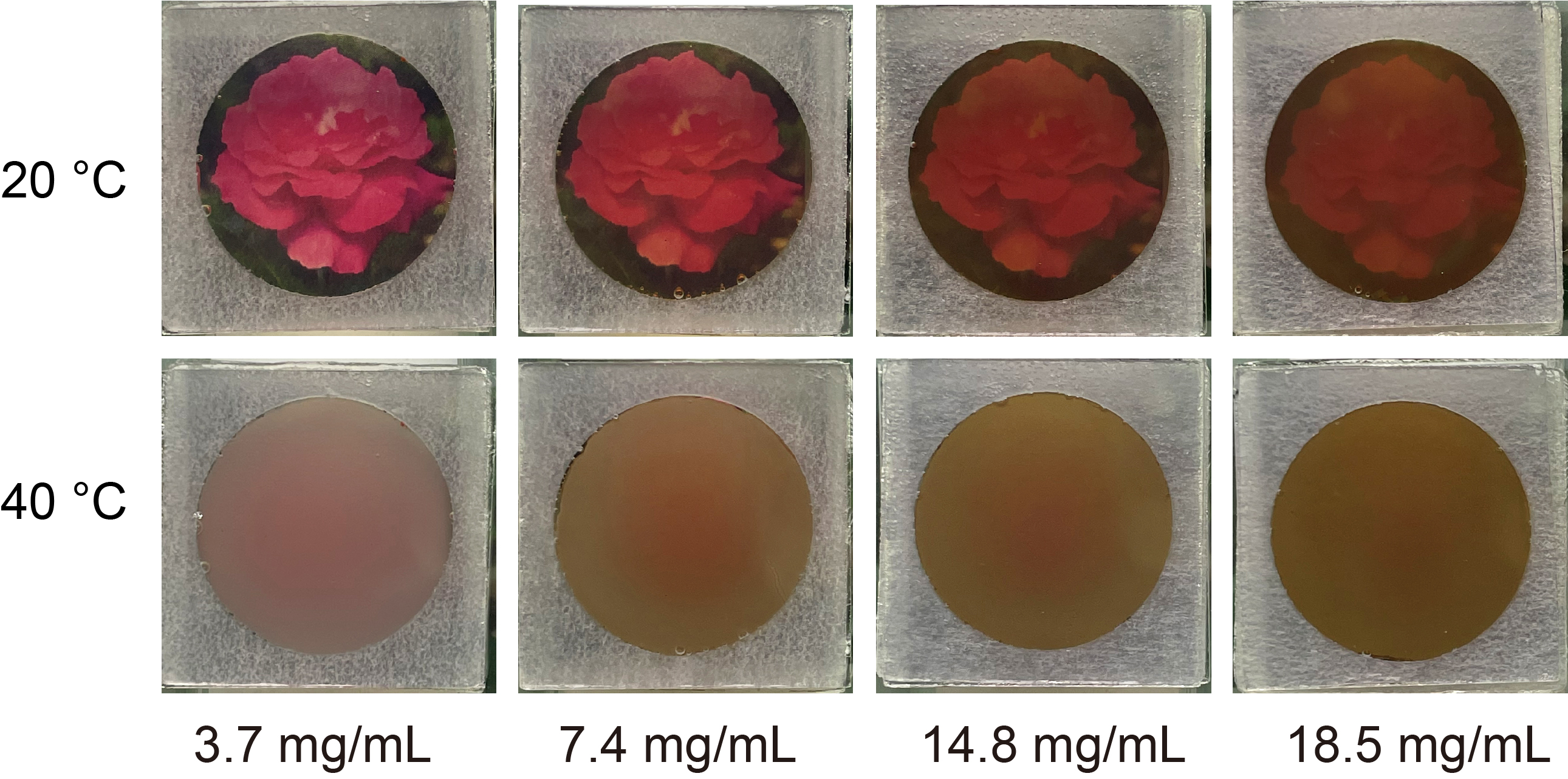


Figure S15. In normal transmittance mode, optical photos for the samples (prepared with different concentration of Fe_3_O_4_@PVP CNCs) at 20 ℃ and 40 ℃, respectively


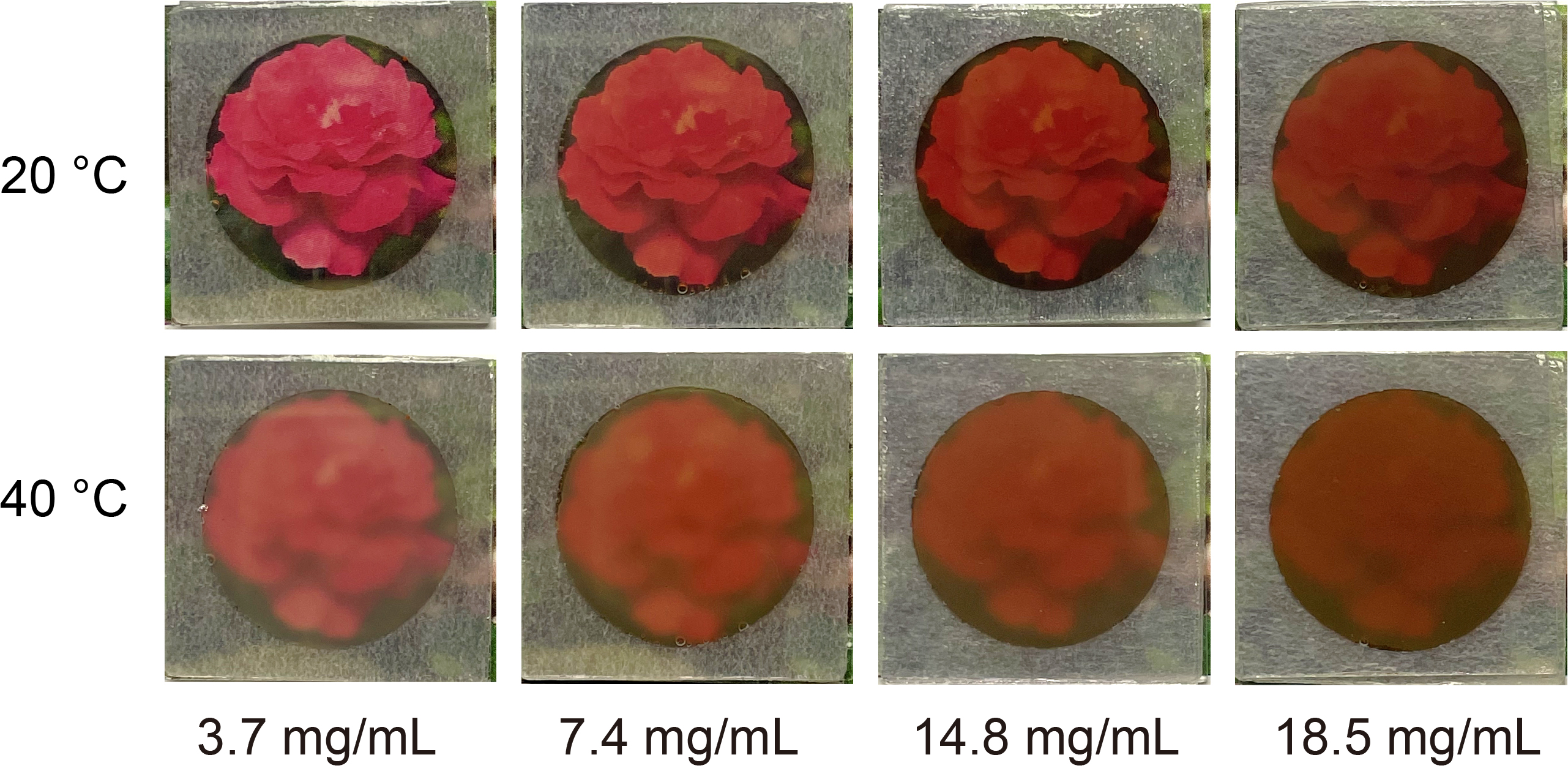


Figure S16. In hemispherical transmittance mode, optical photos for the samples (prepared with different concentration of Fe_3_O_4_@PVP CNCs) at 20 ℃ and 40 ℃, respectively


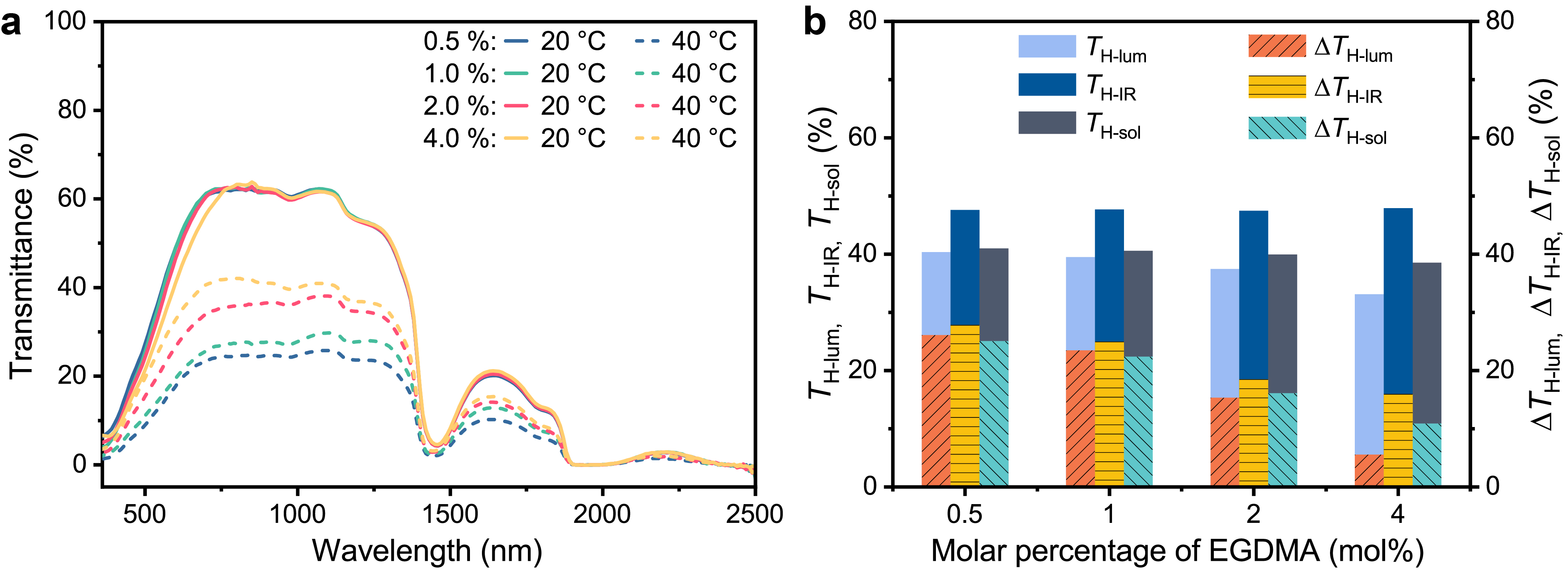


Figure S17. In hemispherical transmittance mode, the transmittance spectra of the samples (prepared with different content of EGDMA) at 20 ℃ (solid line) and 40 ℃ (dashed line), respectively. (b) Comparison of optical performance.


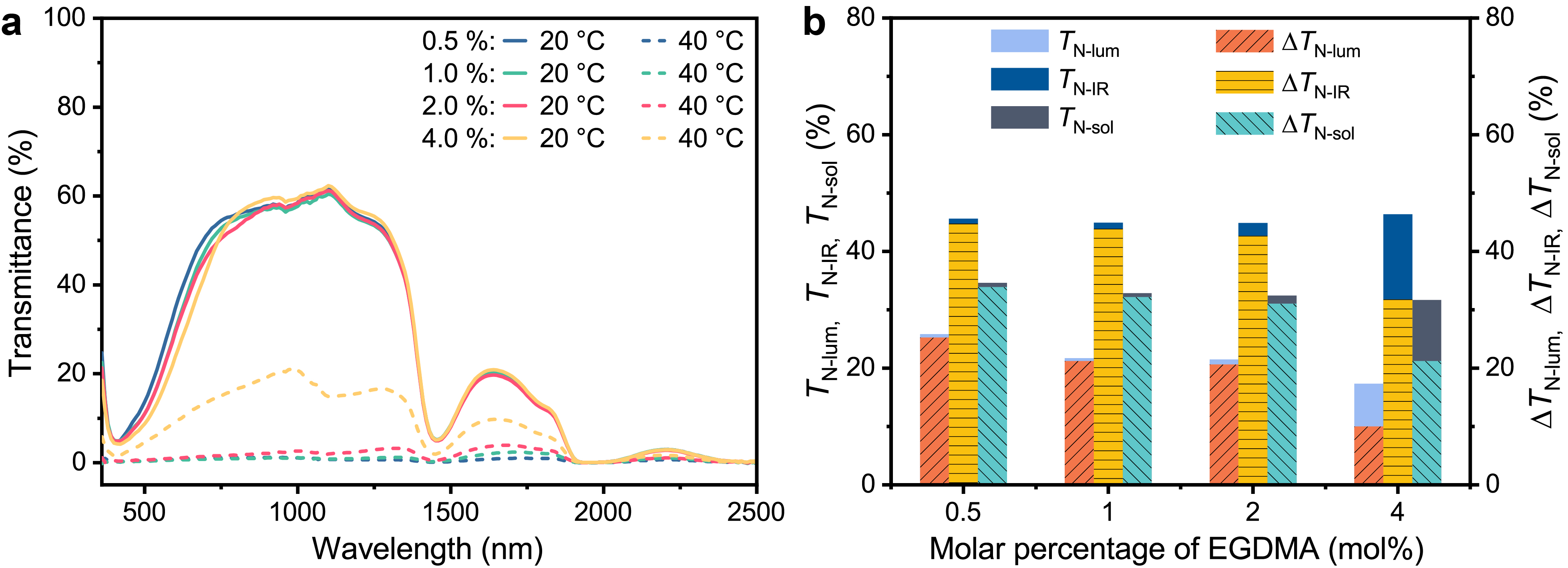


Figure S18. In normal transmittance mode, the transmittance spectra of the samples (prepared with different content of EGDMA) at 20 ℃ (solid line) and 40 ℃ (dashed line), respectively. (b) Comparison of optical performance.


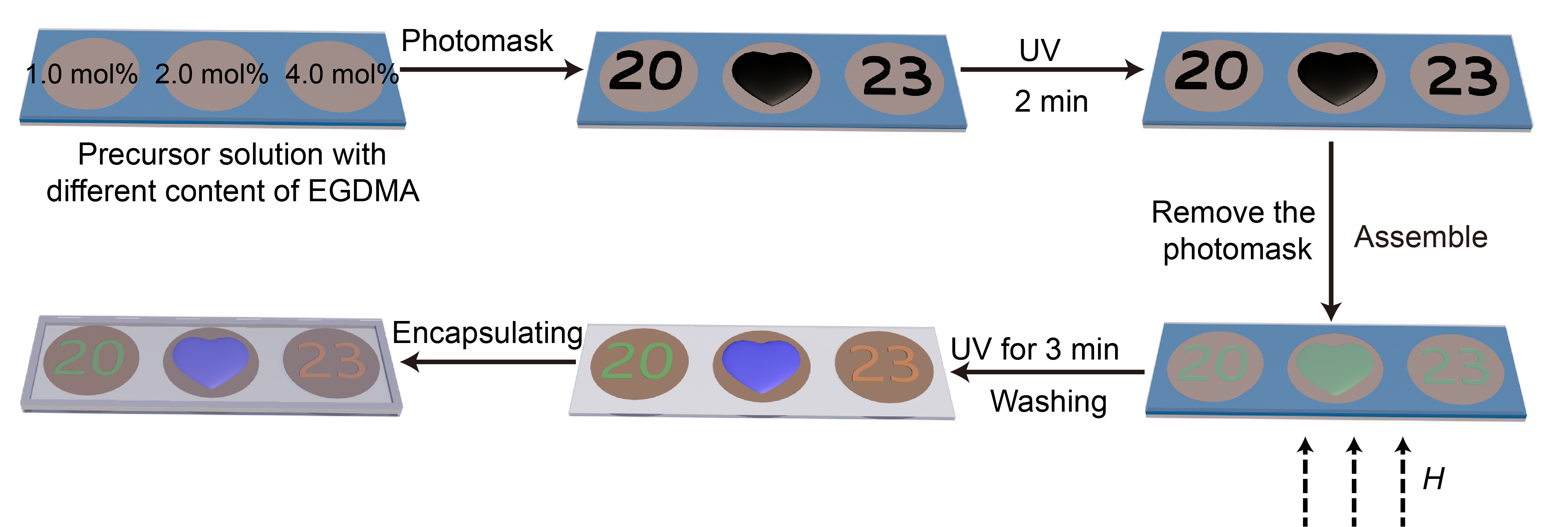


Figure S19. Schematic diagram for the preparation process of the TRPCHF with “20🎔23” pattern.


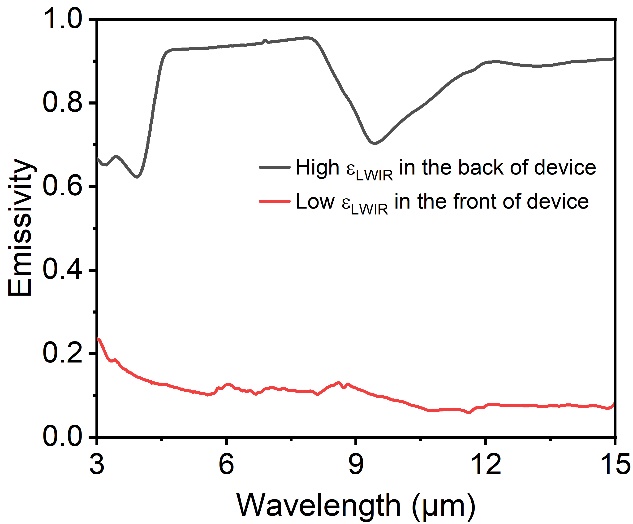


Figure S20. Emissivity spectra for the two sides of the device.
